# Effects of Aortic Valve Replacement on Severe Aortic Stenosis and Preserved Systolic Function: Systematic Review and Network Meta-analysis

**DOI:** 10.1038/s41598-017-05021-9

**Published:** 2017-07-11

**Authors:** Qishi Zheng, Andie H. Djohan, Enghow Lim, Zee Pin Ding, Lieng H. Ling, Luming Shi, Edwin Shih-Yen Chan, Calvin Woon Loong Chin

**Affiliations:** 1 Department of Epidemiology, Singapore Clinical Research Institute, Singapore, Singapore; 2 Cochrane Singapore, Singapore, Singapore; 3 Department of Internal Medicine, National University Hospital, Singapore, Singapore; 4 Duke-NUS Medical School, Singapore, Singapore; 5 Department of Cardiovascular Medicine, National Heart Centre, Singapore, Singapore; 6 Department of Cardiology, National University Heart Center, Singapore, Singapore; 7 Yong Loo Lin School of Medicine, National University of Singapore, Singapore, Singapore

## Abstract

The survival benefits of aortic valve replacement (AVR) in the different flow-gradient states of severe aortic stenosis (AS) is not known. A comprehensive search in PubMed/MEDLINE, Embase, Cochrane Library, CNKI and OpenGrey were conducted to identify studies that investigated the prognosis of severe AS (effective orifice area ≤1.0 cm^2^) and left ventricular ejection fraction ≥50%. Severe AS was stratified by mean pressure gradient [threshold of 40 mmHg; high-gradient (HG) and low-gradient (LG)] and stroke volume index [threshold of 35 ml/m^2^; normal-flow (NL) and low-flow (LF)]. Network meta-analysis was conducted to assess all-cause mortality among each AS sub-type with rate ratio (RR) reported. The effects of AVR on prognosis were examined using network meta-regression. In the pooled analysis (15 studies and 9,737 patients), LF states (both HG and LG) were associated with increased mortality rate (**LFLG**: RR 1.88; 95% CI: 1.43-2.46; **LFHG**: RR: 1.77; 95% CI: 1.16-2.70) compared to moderate AS; and NF states in both HG and LG had similar prognosis as moderate AS (**NFLG**: RR 1.11; 95% CI: 0.81-1.53; **NFHG**: RR 1.16; 95% CI: 0.82-1.64). AVR conferred different survival benefits: it was most effective in NFHG (**RR**_**with AVR**_ **/RR**_**without AVR**_: 0.43; 95% CI: 0.22-0.82) and least in LFLG (**RR**_**with AVR**_ **/RR**_**without AVR**_: 1.19; 95% CI: 0.74-1.94).

## Introduction

The prevalence of calcific aortic stenosis (AS) increases with age, occurring in 3% to 5% of individuals 75 years and above^1^. Contemporary guidelines from the American Heart Association/American College of Cardiology and the European Society of Cardiology recommend aortic valve replacement (AVR) in patients with severe aortic stenosis and evidence of decompensation, either in the form of symptoms or left ventricular systolic dysfunction^2, 3^.

Severe AS is defined by an effective orifice area (EOA) of <1.0 cm^2^, and a mean pressure gradient (MPG) > 40 mmHg or peak aortic jet velocity (V_m_) > 4 m/s^4^. It has recently been proposed that severe AS with preserved systolic function can be further stratified into 4 flow-gradient states: normal flow-low gradient (NFLG), low flow-low gradient (LFLG), normal flow-high gradient (NFHG), and low flow-high gradient (LFHG) severe AS^5^. Paradoxical low flow states (indexed stroke volumes <35 ml/m^2^) account for a proportion of patients with discordant small EOA and low mean pressure gradient (MPG) despite preserved systolic function^6, 7^. First described in 2007, LF with preserved systolic function (paradoxical LF) AS was associated with increased all-cause mortality compared to NF ^8^. Subsequent studies have suggested that paradoxical LG AS may be less severe compared to HG states and a prognosis more akin to moderate disease^9, 10^, yet others have reported that it had the worst prognosis compared to all the other flow-gradient states^5, 11^. Two meta-analyses demonstrated poor prognosis associated with LFLG AS; and prognosis of severe aortic stenosis (regardless of flow-gradient states) improved with AVR compared to conservative management^12, 13^. However, the relative benefits of AVR may be different in patients with different flow-gradient states.

Network meta-analysis is an approach that incorporates evidence from direct comparisons with indirect comparisons that are extracted from multiple studies^14^. Using network meta-analysis, we aimed to assess the prognosis associated with the different flow-gradient states of severe AS and preserved systolic function. We further examined the relative effects of AVR on survival in individuals with different flow-gradient states, with moderate AS as the reference.

## Methods

### Literature Search and Eligibility Criteria

A comprehensive literature search was performed on electronic databases: PubMed/MEDLINE (1946 onwards), Embase (1974 onwards), Cochrane library (1992 onwards), China Knowledge Research Integrated Database (CNKI; 1979 onwards) and OpenGrey (2000 onwards) until 28 February 2017. The specific concepts used in the search strategy were “severe aortic stenosis”, “flow”, “gradient” and “prognosis”. We conducted literature search using Medical Subject Headings (MeSH) or Emtree, and free text terms (see online supplemental data). There were no restrictions on language. All the bibliography listed in review papers and included publications were also checked.

Two investigators (CWLC and QSZ) independently screened for eligible studies based on pre-defined eligibility criteria. Full-text studies that examined the prognosis of severe AS (EOA ≤1.0 cm^2^ stratified by flow and gradient states) with preserved systolic function (LVEF ≥50%) and published after the introduction of the classification system in 2007 were included. We excluded studies that examined prognosis in AS with impaired systolic function (LVEF <50%) and those that did not specify flow-gradient states. Case reports, commentaries and letters-to-editors were also excluded. Flow states were categorized as according to low (<35 ml/m^2^) and normal flow (≥35 ml/m^2^). Low and high MPG were defined by a gradient of <40 mmHg and ≥40 mmHg, respectively. Therefore, 4 subgroups of severe AS were defined: LFLG, NFLG, LFHG and NFHG.

### Data extraction and quality assessment

The following data were extracted from the included studies: (1) study characteristics (publication year, patient population and the type of cohort study); (2) baseline characteristics (mean age, proportion of symptomatic status, number of males/females and proportion of patients with hypertension, coronary artery disease and diabetes); (3) valve severity and flow states; and (4) outcome events (all-cause mortality regardless of AVR). In publications with survival curves, the cumulative survival rates were estimated by digitizing the plots^15, 16^.

The quality of each study was evaluated, using the Quality In Prognosis Studies tool (QUIPS tool), by two independent investigators (QSZ and LMS)^17^. Six domains (study participation study attrition, prognostic factor measurement, outcome measurement, study confounding and statistical analysis and reporting) were evaluated to assess the risk of bias in prognostic studies. For each of the 6 domains in the QUIPS tool, responses to the signaling questions are taken together to arrive at the judgment of “High”, “Moderate” or “Low” risk of bias. Any disagreement in quality assessment was resolved by discussion and consensus.

### Statistical analysis

A network geometry was constructed based on the included studies for subgroups of severe AS. Each node represented a subgroup and its size was weighted by the number of subjects of each subgroup. The connecting line between two nodes meant direct comparison was existed and its thickness was determined by the number of studies included.

Network meta-analysis, comparing the prognosis of the different severe AS sub-groups, was performed using a multivariate meta-regression model with random-effects, adopting a frequentist approach^18, 19^. The model allows for the inclusion of potential covariates, and accounts for the correlations from multi-arm trials, and rate ratio (RR) of each subgroup was estimated^20^.

To rank the prognosis for all the groups, we used surface under the cumulative ranking (SUCRA) values^21^. Rank probabilities of all the groups were first estimated under a Bayesian framework. A step function was then applied to summarize the cumulative ranking for estimating the SUCRA values of each group, ranging from 0 to 1. Thus, a large SUCRA value indicated a better prognosis.

Meta-regression was used to investigate the effects of AVR on survival in the different flow-gradient patterns of AS^19^. The node-splitting approach and inconsistency model were used to test the consistency assumption^22^. The former method involved fitting a series of node-splitting models, with one model for each sub-group pairing for which there was direct and indirect evidence^23^. The latter method first fits an inconsistency model and then conduct a global Wald test to check whether there is significant inconsistency exists^14^.

The network meta-analyses and meta-regression were implemented by Stata/MP 13 with network and network graphs package^14, 24, 25^.

## Results

### Study characteristics and network geometry

**Figure 1:**
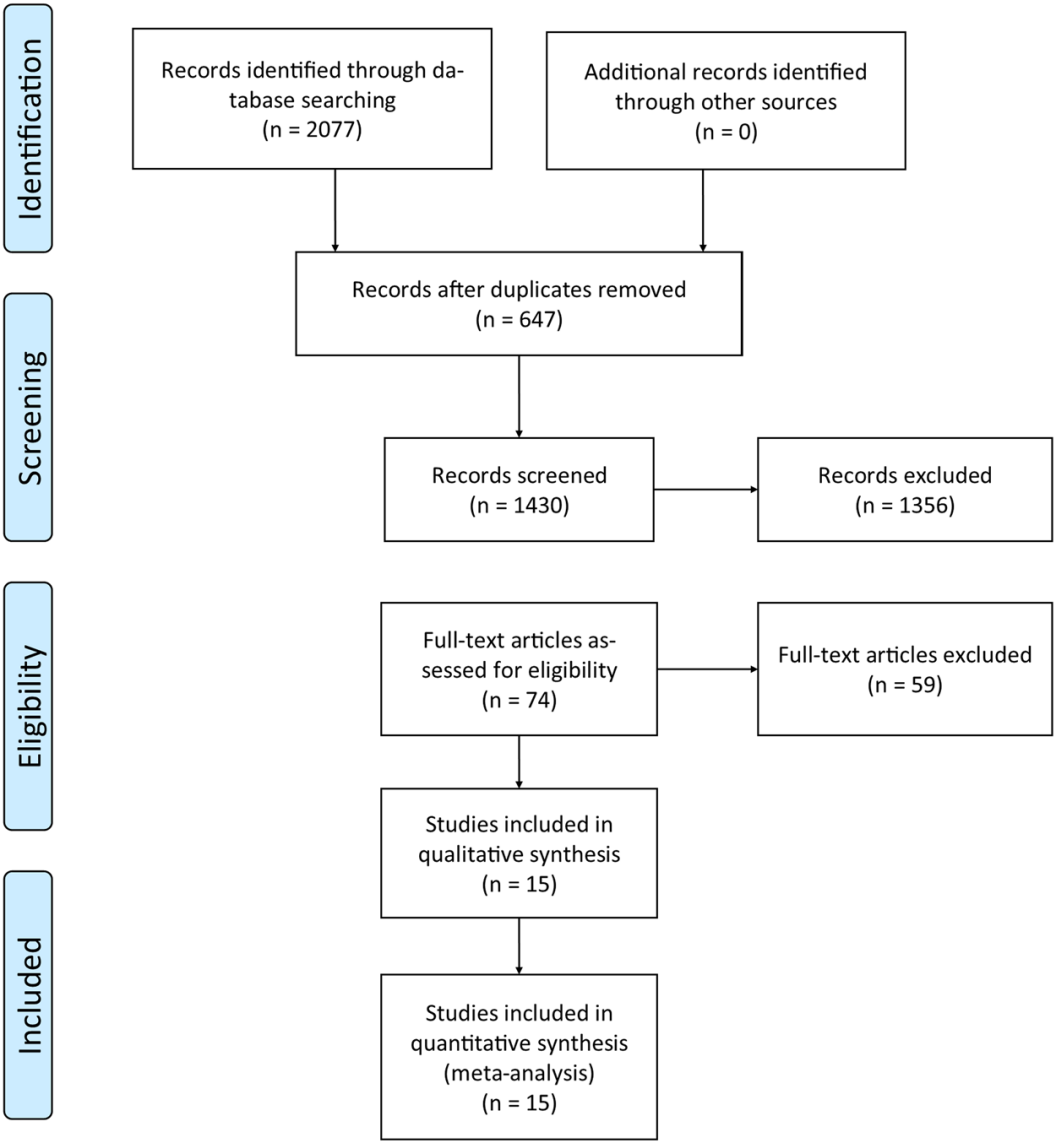
PRISMA flowchart of study selection. The systematic review and meta-analysis was conducted according to the guidelines recommended by PRISMA^48^.

From 1,274 potential studies identified from the initial search, 15 cohort studies (n = 9,737 individuals) satisfied inclusion/exclusion criteria and were included in this meta-analysis^10, 11, 26–47^ (Fig. 1; Table 1). No randomized controlled studies were found. The mean age was 76±6 years and 43% were males. Of note, 29%, 38% and 63% of individuals had diabetes, coronary artery disease and hypertension respectively, with the duration of follow-up varied between 0.8 and 4.9 years (Tables 1 and 2). Twelve studies examined the effects of AVR on prognosis.

**Table 1:** Study characteristics.

Study name, Year	Comparison pairs	Age (mean, SD)	Gender (Male)	Diabetes	CAD	HTN	SYM	AVR	Median follow-up (Year)	Cohort study design
Clavel 2012	LFLG (187), NFHG (187), MAS (187)	69, 13.3	339 (60%)	132 (24%)	288 (51%)	395 (70%)	397 (71%)	307 (55%)	4.2	Retrospective
Eleid 2013	LFLG (53), NFHG (1249), NFLG (352), LFHG (50)	78, 11.9	753 (44%)	722 (42%)	472 (28%)	1250 (73%)	1193 (70%)	1121 (66%)	2.3	Retrospective
Fan 2015	LFLG (19), NFHG (59), NFLG (30), LFHG (80)	55, 11.4	99 (53%)	8 (4.3%)	NA	36 (19%)	111 (59%)	188 (100%)	4.9	Mixed
Hermann 2011	LFLG (11), MAS (17)	71, 7.4	11 (39%)	6 (21%)	NA	24 (86%)	12 (43%)	11 (39%)	0.75	Prospective
Kamperidis 2014	LFLG (48), NFLG (86)	76, 10	67 (50%)	34 (25%)	83 (62%)	98 (73%)	48 (36%)	134 (100%)	1.8	Retrospective
Maes 2014	LFLG (115), NFLG (90)	78, 10.2	145 (42%)	77 (22%)	73 (21%)	270 (77%)	129 (37%)	92 (26%)	2.3	Prospective
Maor 2014	LFLG (136), NFLG (273)	78, 11	172 (42%)	132 (33%)	164 (40%)	245 (60%)	NA	94 (23%)	2.92	Mixed
Mehrotra 2013	LFLG (38), NFLG (75), MAS (70)	78, 9.4	86 (47%)	37 (20%)	63 (34%)	161 (88%)	21 (12%)	0 (0%)	3	Retrospective
Melis 2013	LFLG (42), NFHG (169), NFLG (98), LFHG (54)	76.8	177 (49%)	104 (29%)	136 (38%)	270 (74%)	NA	216 (59%)	2.1	Retrospective
Mohty 2013	LFLG (99), NFHG (386), NFLG (172), LFHG (111)	74, 8	445 (58%)	161 (21%)	353 (46%)	476 (62%)	684 (89%)	699 (91%)	4.6	Retrospective
O’Sullivan 2013	LFLG (85), LFHG (208)	83, 5.2	118 (40%)	82 (28%)	164 (56%)	247 (84%)	199 (68%)	293 (100%)	1	Retrospective
Romero 2014	LFLG (776), MAS (2958)	78, 12.3	1324 (31%)	1048 (25%)	1684 (40%)	2072 (49%)	NA	432 (10%)	2.3	Retrospective
Schewel 2015	LFLG (104), NFHG (289)	81, 6.8	149 (38%)	116 (30%)	218 (55%)	345 (88%)	352 (90%)	393 (100%)	1	Prospective
Tribouilloy 2015	LFLG (57), NFLG (85), MAS (420)	77, 4.1	306 (54%)	179 (32%)	176 (31%)	421 (75%)	74 (13%)	0 (0%)	3.25	Retrospective
Yamashita 2015	NFLG (61), MAS (151)	76, 9	93 (44%)	73 (34%)	76 (36%)	164 (77%)	79 (37%)	0 (0%)	1.3	Retrospective

MAS: moderate aortic stenosis; LFLG: low-flow low-gradient; NFLG: normal-flow low-gradient; IFLG: indeterminate-flow low-gradient; LFHG: low-flow high-gradient; NFHG: normal-flow high-gradient; IFHG: indeterminate-flow high-gradient; CAD: coronary artery disease; HTN: hypertension; SYM: symptoms present; AVR: aortic valve replacement.

**Table 2:** Baseline Characteristics Stratified by Flow-Gradient States.

Characteristics	MAS (n = 3,803)	LFLG (n = 1,762)	NFLG (n = 1,330)	LFHG (n = 512)	NFHG (n = 2,330)
Age (SD), years	77 (2.2)	79 (3.6)	77 (4.4)	76 (8.8)	75 (4.8)
Females, %	63	62	56	55	43
Diabetes Mellitus, %	26	29	33	27	31
Coronary artery disease, %	36	49	40	45	33
Hypertension, %	53	67	73	67	69
Symptoms, %	26	67	49	75	78
Atrial fibrillation, %	13	41	16	21	16
Dyslipidemia, %	28	40	54	54	46
Smoking, %	8	7	14	11	12
Aortic valve replacement, %	5	35	40	91	81

Of the 9,737 individuals in the studies, 39% (n = 3,803), 29% (n = 2,842) and 32% (n = 3,092) of individuals had moderate, HG and LG severe AS, respectively. Amongst the individuals with LG AS, 57% (n = 1,762) and 43% (n = 1,330) had low and normal flow, respectively. Conversely, significantly more individuals with HG had normal flow (82%; n = 2,330) compared to low flow states (18%; n = 512). Moderate AS was used as the reference group for comparison in this study.

**Figure 2:**
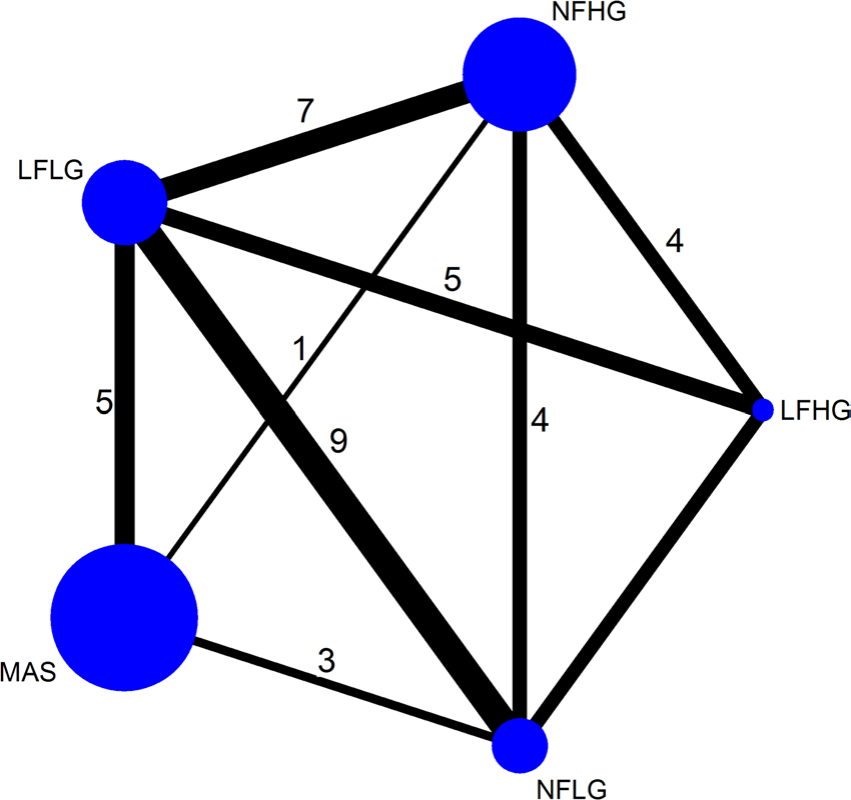
Network geometry of the different flow-gradient states of severe aortic stenosis and preserved systolic ejection fraction.

The quality of all the studies demonstrated low to moderate risk of bias in the six domains assessed (Table 3). The network geometry was constructed (Fig. 2).

**Table 3:** Risk of bias assessment for included studies.

	Study Participation	Study Attrition	Prognostic Factor Measurement	Outcome Measurement	Study Confounding	Statistical Analysis and Reporting
Clavel 2012	Low	Low	Low	Low	Low	Low
Eleid 2013	Low	Low	Low	Low	Low	Low
Fan 2015	Moderate	Low	Low	Low	Low	Moderate
Hermann 2011	Low	Low	Low	Low	Low	Moderate
Kamperidis 2014	Low	Moderate	Low	Low	Low	Low
Maes 2014	Low	Low	Low	Low	Low	Low
Maor 2014	Low	Low	Low	Low	Low	Low
Mehrotra 2013	Moderate	Low	Low	Low	Low	Low
Melis 2013	Moderate	Low	Low	Low	Low	Low
Mohty 2013	Low	Low	Low	Low	Low	Low
O’Sullivan 2013	Low	Low	Low	Low	Low	Low
Romero 2014	Moderate	Low	Low	Low	Low	Low
Schewel 2015	Low	Low	Low	Low	Low	Low
Tribouilloy 2015	Low	Low	Low	Low	Low	Low
Yamashita 2015	Low	Low	Low	Low	Low	Low

### Network meta-analysis results

**Figure 3:**
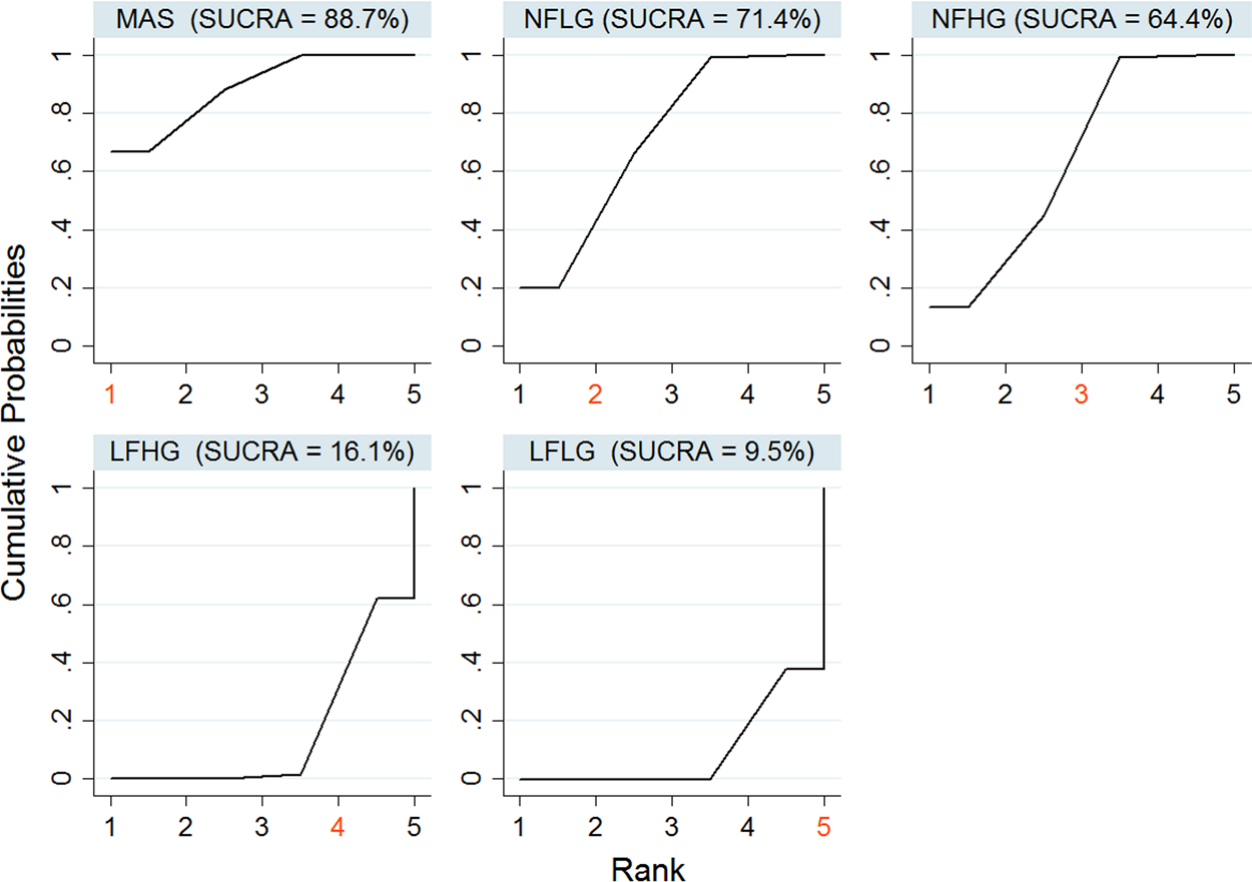
Cumulative probability curves for prognosis for each group with SUCRA values.

In both HG and LG AS, low flow states were associated with increased all-cause mortality (**LFLG**: RR 1.88; 95% CI: 1.43 to 2.46; **LFHG**: RR: 1.77; 95% CI: 1.16 to 2.70; Table 4). Of note, both low flow subgroups had similar mortality rate (the ratio of LFHG and LFLG RRs was 0.94; 95% CI: 0.67 to 1.33). Conversely, normal flow states in both HG and LG had similar prognosis compared to moderate AS (**NFLG**: RR 1.11; 95% CI: 0.81 to 1.53; **NFHG**: RR 1.16; 95% CI: 0.82 to 1.64; Table 4). Based on the SUCRA values of each subgroup, the prognosis of the different flow-gradient states ranked from best to worst would be: **NFLG, NFHG, LFHG and LFLG** (Fig. 3). Results from both node-splitting method and inconsistency model showed no evidence of violating the assumption of consistency between direct and indirect comparison (P = 0.507; ﻿Fig. ﻿4).

**Table 4:** Rate ratios of all-cause mortality from network meta-analysis with 95% confidence interval.

	MAS	LFLG	NFLG	LFHG	NFHG
MAS	1	1.88 (1.43,2.46)	1.11 (0.81,1.53)	1.77 (1.16,2.70)	1.16 (0.82,1.64)
LFLG	0.53 (0.41, 0.70)	1	0.59 (0.46,0.76)	0.94 (0.67,1.33)	0.62 (0.48,0.80)
NFLG	0.90 (0.65, 1.23)	1.69 (1.32, 2.17)	1	1.59 (1.09,2.31)	1.04 (0.78,1.40)
LFHG	0.56 (0.37, 0.86)	1.06 (0.75, 1.49)	0.63 (0.43, 0.92)	1	0.66 (0.46,0.93)
NFHG	0.86 (0.61, 1.22)	1.61 (1.25, 2.08)	0.96 (0.71, 1.28)	1.52 (1.08, 2.17)	1

MAS: moderate aortic stenosis; LFLG: low-flow low-gradient; NFLG: normal-flow low-gradient; LFHG: low-flow high-gradient; NFHG: normal-flow high-gradient.

All the RRs were presented using the row sub-type as the reference group.

**Figure 4:**
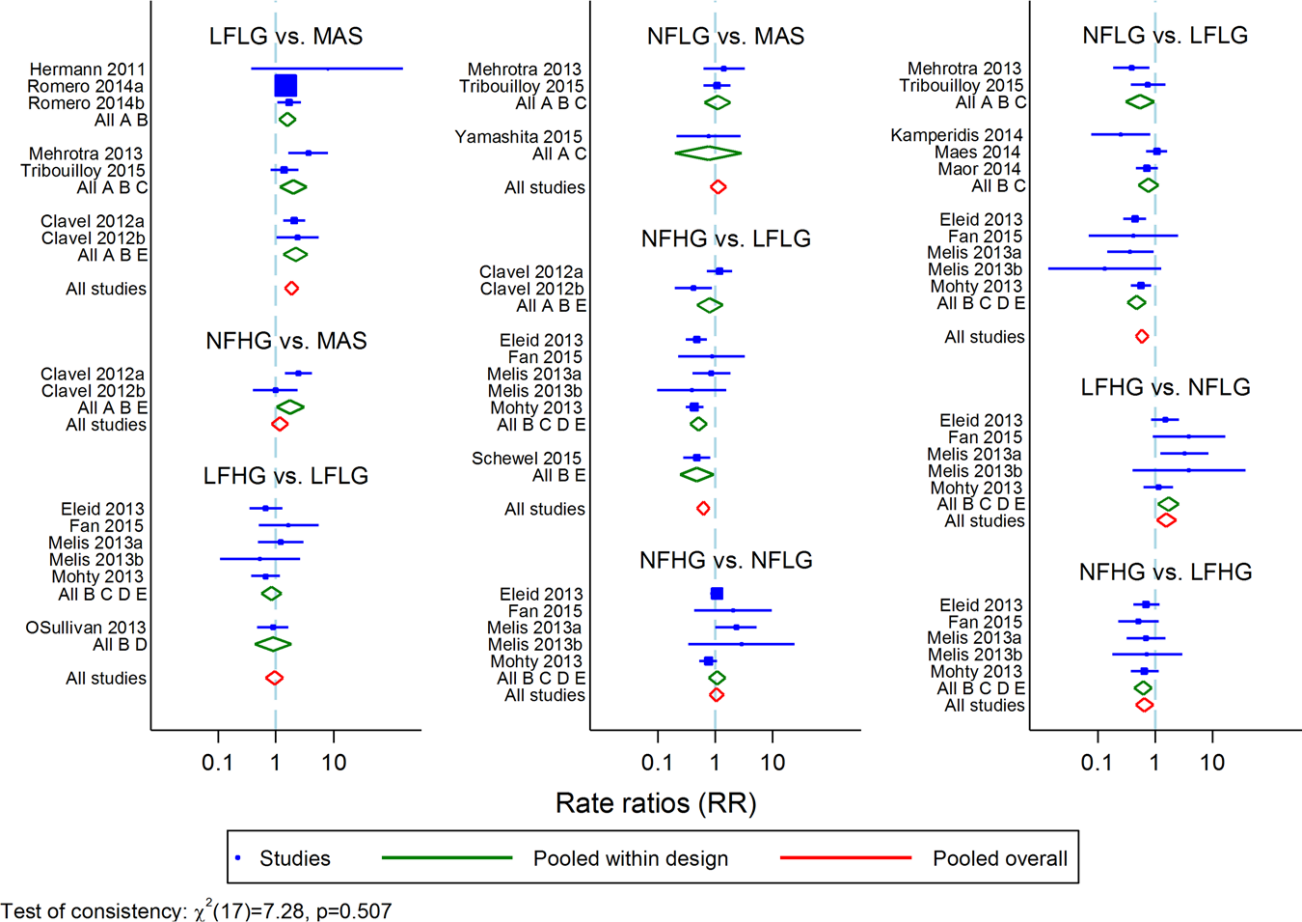
Forest plots with results from consistency (red diamond) and inconsistency (green diamond) model.

### Comparative effects of AVR on survival

**Figure 5:**
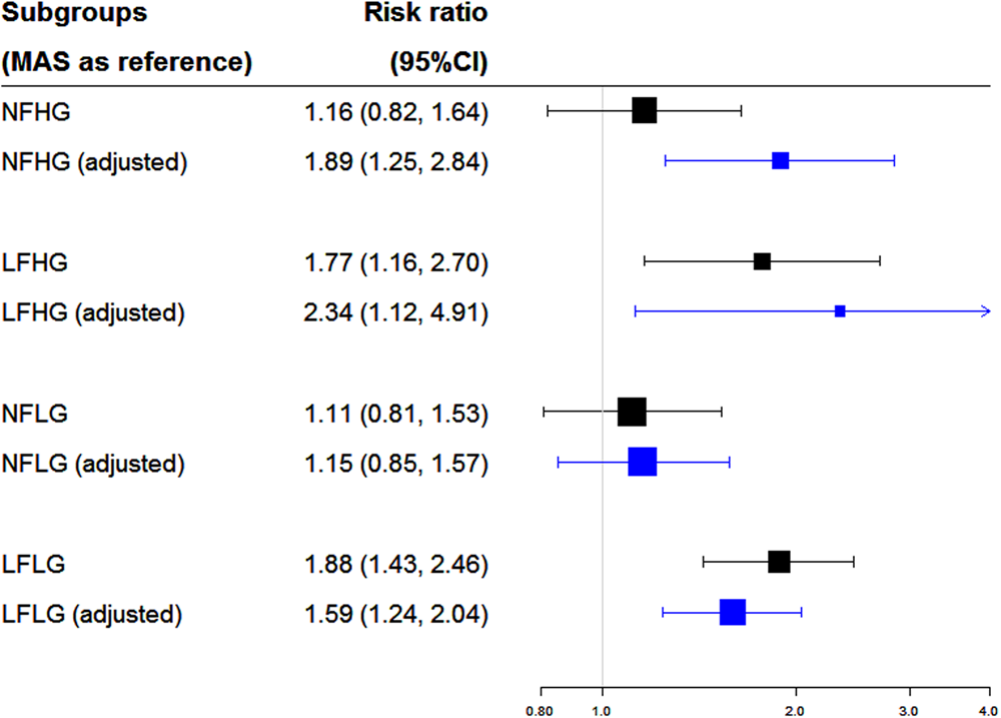
Effects of AVR on the different flow-gradient states of severe aortic stenosis.

To examine the relative effects of AVR on survival in individuals with different flow-gradient states, we compared the RRs before and after adjusting for AVR (Fig. 5). In NFHG severe AS, the RR improved from 1.89 (95% CI: 1.25 to 2.84) to 1.16 (95% CI: 0.82 to 1.64) with AVR (**RR**_**with AVR**_ **/RR**_**without AVR**_ **:** 0.43; 95% CI: 0.22 to 0.82). Whilst AVR conferred a survival benefit for NFHG, it may not benefit all patients with LFLG equally. The mortality RR of LFLG increased from 1.59 (95% CI: 1.24 to 2.04) to 1.88 (95% CI: 1.43 to 2.46) with AVR (**RR**_**with AVR**_ **/RR**_**without AVR**_ **:** 1.19; 95% CI: 0.74 to 1.94; Fig. 5). There was a trend towards benefit in severe AS patients with LFHG (**RR**_**with AVR**_ **/RR**_**without AVR**_: 0.63; 95% CI: 0.24 to 1.67) and NFLG (**RR**_**with AVR**_ **/RR**_**without AVR**_: 0.87; 95% CI: 0.45 to 1.68).

**Figure 6:**
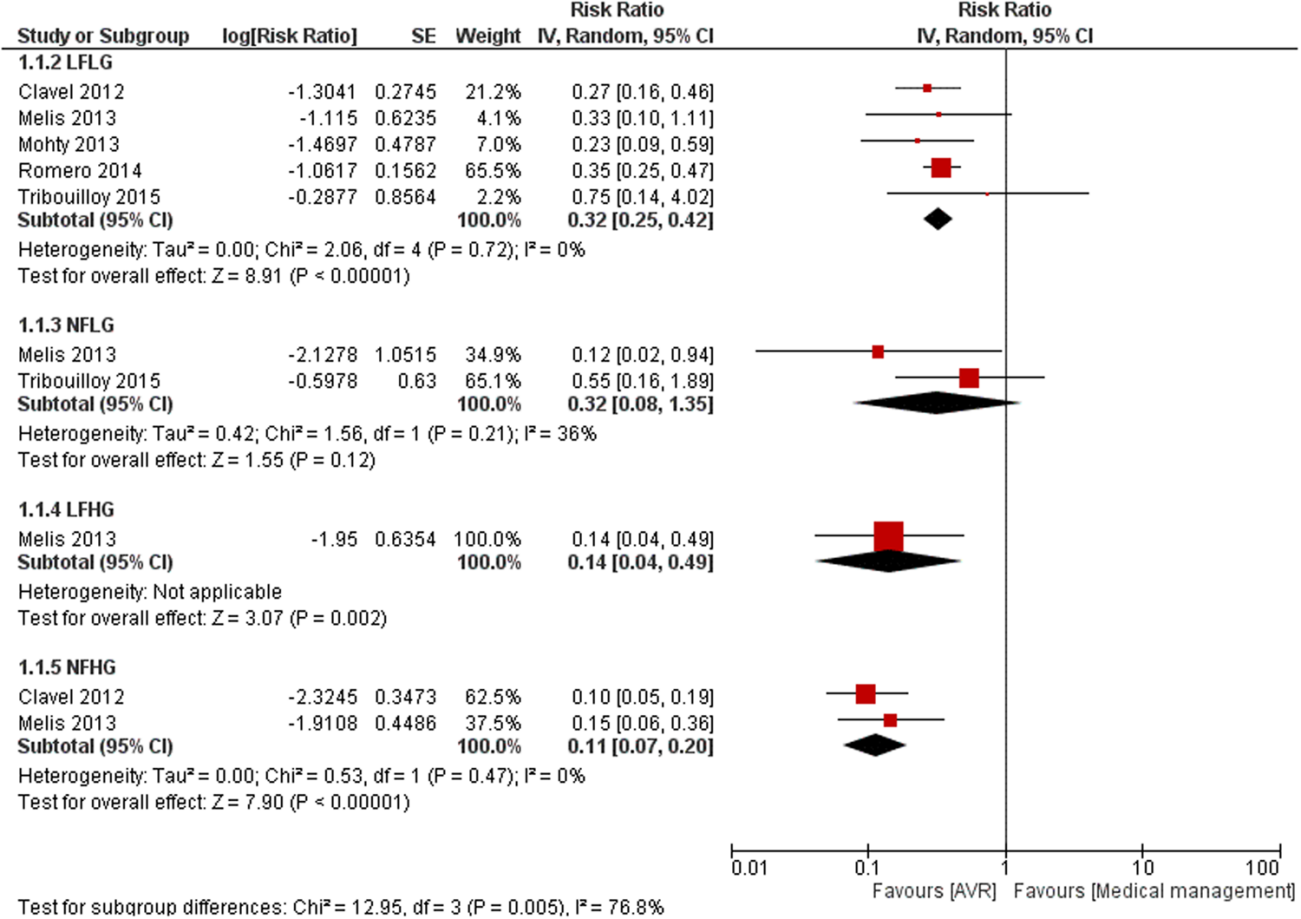
Direct comparisons of the effects of AVR versus medical therapy on the different flow-gradient states.

Similar findings were observed when traditional pair-wise comparisons were performed (Fig. 6). Compared to medical therapy, AVR improved outcomes in all flow-gradient states of severe AS but with different relative benefits. NFHG severe AS derived the most relative benefits from AVR (RR: 0.11; 95% CI: 0.07 to 0.20) compared to LFLG severe AS (RR: 0.32; 95% CI: 0.25 to 0.42).

## Discussion

Our network meta-analysis investigated the prognostic effects of AVR on the different flow-gradient states of severe AS. Overall, we demonstrated that the risk of all-cause mortality in severe AS was associated with low flow states, and not valvular gradients. Normal flow states had similar prognosis to moderate AS. Moreover, the relative survival benefits conferred by AVR were different in patients with severe AS, depending on flow-gradient states. Individuals with NFHG derived the greatest survival benefit from AVR among all the subgroups of severe AS.

The traditional pair-wise meta-analysis can only synthesize evidence from studies with direct comparisons of two arms being tested. However, it is difficult to rank the prognosis of each subgroup of severe AS, as such direct evidence is limited, i.e. only 4 out of 15 studies included all the flow-gradient states of interest (LFLG, NFLG, LFHG and NFHG). Therefore, the traditional pairwise approach is not ideal to comprehensively investigate the prognosis of the different flow-gradient states from the existing evidence. By synthesizing direct and indirect evidence using network meta-analysis, we were able to examine the prognosis of all the different flow-gradient states in severe AS.

In our study, low flow states (not valvular gradient) in severe AS were associated with an increased risk of all-cause mortality. Compared to two recent meta-analyses that have combined flow states in HG^12, 13^, we demonstrated that HG severe AS can further be risk-stratified based on flow patterns: the prognosis of NFHG was similar to moderate AS, and better than LFHG. These findings are consistent with a recent study that demonstrated a continuous association between adverse events and reduced stroke volumes in patients with AS and preserved systolic function, independent of valvular gradient^49^. The complex interaction of the valve, comorbid medical conditions and the myocardium account for the different flow-gradient states in AS^50, 51^. In the presence of preserved systolic function, low flow states can be present in individuals with significant myocardial hypertrophy. Other factors contributing to low flow states such as atrial fibrillation (41% in those with LFLG in the pooled studies), hypertension and concomitant valvular heart disease (mitral stenosis or mitral regurgitation) can also result in increased mortality.

Although patients with severe AS (regardless of flow or gradient) have improved outcomes with AVR compared to conservative management^12^, it is possible AVR would confer different survival benefits since the prognosis varied across the flow-gradient states. Our study adds novelty by demonstrating the relative survival benefits of AVR vary with flow-gradient states. Patients with classically severe (NFHG) AS derived the most benefit from AVR whilst the risk of mortality was not completely modified by AVR in those with paradoxical LFLG severe AS. These findings were confirmed using traditional pair-wise comparisons. The role of AVR in the other flow-gradient states should be interpreted with caution. There was a trend towards benefit in severe AS patients with LFHG and NFLG. The wide confidence limits may be explained by the relatively smaller number of individuals compared to other flow-gradient states.

### Clinical Implications

These findings have important clinical implications. Paradoxical LG severe AS may be conceptualized as a heterogeneous condition akin to heart failure with preserved ejection fraction (HFpEF)^51^. Both conditions share similar clinical characteristics and mechanisms of cardiac adaptation^12, 51, 52^. Similar to paradoxical LG severe AS, a high proportion of adverse events are related to non-cardiovascular comorbidities in HFpEF^52^. Patients with paradoxical LFLG severe AS may benefit from AVR, as currently recommended by both the ACC/AHA and ESC (Class IIa)^2, 3^. For reasons discussed above, AVR may not completely ameliorate the risks in those with paradoxical LG severe AS. Therefore, it is crucial to confirm severity of disease and improve risk stratification using either imaging (stress echocardiography, aortic valve CT calcium score and cardiovascular magnetic resonance) or biochemical markers^53–59^. However, the benefits of AVR in at-risk individuals identified using such a strategy will need to be carefully tested in a clinical trial.

### Study Limitations

Due to incomplete data we were unable to assess the prognosis on cardiovascular mortality. The adjustment of AVR in the current analysis can only be conducted at the study-level, which might not accurately reflect the prognosis of the individual patient. In the absence of any randomized controlled studies, the effects of AVR may be confounded by potential biases inherent to the observational nature of the studies. For example, AVR in individuals with LFLG may be delayed because of an uncertainty in diagnosis and management. Potential study inconsistency may affect the validity of the network meta-analysis. However, we have carefully assessed for any potential violation of the consistency assumption. Similar estimates from direct and indirect comparisons were demonstrated and formal statistical testing did not reveal violation of consistency.

## Conclusions

Low flow states (regardless of valvular gradient) were associated with increased all-cause mortality amongst individuals with severe AS. Of all the flow-gradient states, NFHG was associated with the greatest survival benefit from AVR. Properly designed randomized studies are crucially needed to clarify the role of AVR in individuals with LFLG severe AS.

## Electronic supplementary material

Supplementary Information
