# Supplementary material for: Effects of Aortic Valve Replacement on Severe Aortic Stenosis and Preserved Systolic Function: Systematic Review and Network Meta-analysis
Source: Sci Rep. 2017 Jul 11;7:5092. doi: 10.1038/s41598-017-05021-9 (PMC5505951; doi:10.1038/s41598-017-05021-9)
Supplement: Supplementary file 1 — Supplementary Information [file 41598_2017_5021_MOESM1_ESM.doc]

**Effects of Aortic Valve Replacement on Severe Aortic Stenosis and Preserved Systolic Function: Systematic Review and Network Meta-analysis**

**ONLINE SUPPLEMENTAL DATA**

**Search strategies**

**MEDLINE/PubMed**

#1 Aortic Valve Stenosis[MeSH Terms] OR Aortic Stenosis[Title/Abstract] OR Valve Stenosis[Title/Abstract]

#2 flow[Title/Abstract] OR output[Title/Abstract] OR gradient[Title/Abstract] OR pressure[Title/Abstract]

#3 low[Title/Abstract] OR lower[Title/Abstract]

#4 Prognosis[MeSH Terms] OR Prognosis[Title/Abstract] OR prognostic[Title/Abstract]

#5 Vital Statistics[MeSH Terms] OR mortality[Title/Abstract] OR mortalities[Title/Abstract] OR morbidity[Title/Abstract] OR morbidities[Title/Abstract]

#6 Survival Analysis[MeSH Terms] OR Death[MeSH Terms] OR Survive[Title/Abstract] OR Survival[Title/Abstract] OR Death[Title/Abstract] OR Dead[Title/Abstract]

#7 #4 OR #5 OR #6

#8 #1 AND #2 AND #3 AND #7

**Embase**

#1 'aortic valve stenosis'/exp OR (aortic:ta,ab,ti AND stenosis:ta,ab,ti OR valve:ta,ab,ti AND stenosis:ta,ab,ti)

#2 flow:ta,ab,ti OR output:ta,ab,ti OR gradient:ta,ab,ti OR pressure:ta,ab,ti

#3 low:ta,ab,ti OR lower:ta,ab,ti

#4 'prognosis'/exp OR prognosis:ta,ab,ti OR prognostic:ta,ab,ti

#5 'vital statistics'/exp OR mortality:ta,ab,ti OR mortalities:ta,ab,ti OR morbidity:ta,ab,ti OR morbidities:ta,ab,ti

#6 'survival analysis'/exp OR survive:ta,ab,ti OR survival:ta,ab,ti

#7 'death'/exp OR death:ta,ab,ti OR dead:ta,ab,ti

#8 #4 OR #5 OR #6 OR #7

#9 #1 AND #2 AND #3 AND #8

**Cochrane Library**

#1 Aortic Stenosis:ti,ab,kw or Valve Stenosis:ti,ab,kw

#2 MeSH descriptor: [Aortic Valve Stenosis] explode all trees

#3 flow:ti,ab,kw or OUTPUT:ti,ab,kw or gradient:ti,ab,kw or pressure:ti,ab,kw

#4 LOW:ti,ab,kw or LOWER:ti,ab,kw

#5 Prognosis or prognostic or mortality or mortalities or morbidity or morbidities or Survive or Survival Death or Dead:ti,ab,kw (Word variations have been searched)

#6 MeSH descriptor: [Prognosis] explode all trees

#7 MeSH descriptor: [Vital Statistics] explode all trees

#8 MeSH descriptor: [Survival Analysis] explode all trees

#9 MeSH descriptor: [Death] explode all trees

#10 #1 or #2

#11 #5 or #6 or #7 or #8 or #9

#12 #10 and #11 and #3 and #4

**CNKI**

#1 SU=主动脉狭窄 OR SU=主动脉瓣狭窄

#2 SU=压力阶差 OR SU=压差 OR SU=压力差 OR SU=每搏输出量 OR SU=搏出量 OR SU=搏量

#3 #1 AND #2
